# 7,8-Dihydroxy Efavirenz Is Not as Effective in CYP46A1 Activation In Vivo as Efavirenz or Its 8,14-Dihydroxy Metabolite

**DOI:** 10.3390/ijms25042242

**Published:** 2024-02-13

**Authors:** Natalia Mast, Yong Li, Irina A. Pikuleva

**Affiliations:** Department of Ophthalmology and Visual Science, Case Western Reserve University, Cleveland, OH 44106, USA; nvm2@case.edu (N.M.); yxl665@case.edu (Y.L.)

**Keywords:** efavirenz, CYP46A1, brain, Alzheimer’s disease, cholesterol, (*rac*)-7,8dihydroxy efavirenz, (*rac*)-8,14dihydroxy efavirenz

## Abstract

High dose *(S)*-efavirenz (EFV) inhibits the HIV reverse transcriptase enzyme and is used to lower HIV load. Low-dose EFV allosterically activates CYP46A1, the key enzyme for cholesterol elimination from the brain, and is investigated as a potential treatment for Alzheimer’s disease. Simultaneously, we evaluate EFV dihydroxymetabolites for in vivo brain effects to compare with those of *(S)*-EFV. We have already tested (*rac*)-8,14dihydroxy EFV on 5XFAD mice, a model of Alzheimer’s disease. Herein, we treated 5XFAD mice with (*rac*)-7,8dihydroxy EFV. In both sexes, the treatment modestly activated CYP46A1 in the brain and increased brain content of acetyl-CoA and acetylcholine. Male mice also showed a decrease in the brain levels of insoluble amyloid β_40_ peptides. However, the treatment had no effect on animal performance in different memory tasks. Thus, the overall brain effects of (*rac*)-7,8dihydroxy EFV were weaker than those of EFV and (*rac*)-8,14dihydroxy EFV and did not lead to cognitive improvements as were seen in treatments with EFV and (*rac*)-8,14dihydroxy EFV. An in vitro study assessing CYP46A1 activation in co-incubations with EFV and (*rac*)-7,8dihydroxy EFV or (*rac*)-8,14dihydroxy EFV was carried out and provided insight into the compound doses and ratios that could be used for in vivo co-treatments with EFV and its dihydroxymetabolite.

## 1. Introduction

Cholesterol cannot not cross the blood-brain barrier. Therefore, cholesterol homeostasis in the brain is independent from that in the whole body; it mainly relies on in situ biosynthesis and elimination via the formation of 24-hydroxycholesterol (24HC) [1]. The latter is catalyzed by CYP46A1 (cytochrome P450 46A1), a CNS-specific enzyme residing under normal conditions in the neurons of the brain and retina [2,3,4]. CYP46A1 plays a key role in the brain cholesterol homeostasis [5,6], which is disturbed in many neurodegenerative and non-neurodegenerative diseases [7]. Hence, studies on animals, in which CYP46A1 activity was modulated either pharmacologically or by gene delivery/silencing showed that CYP46A1 activation is beneficial in the models of Alzheimer’s (AD), Huntington’s, Nieman-Pick type C, and Machao-Joseph diseases as well as amyotrophic lateral sclerosis, glioblastoma, depression, and prion infections [8,9,10,11,12,13,14,15,16,17,18,19]. CYP46A1 inhibition was beneficial in mouse models and a subset of patients with certain seizure types [20,21,22,23,24], whereas CYP46A1 activation by EFV was required to reduce seizure frequency in a mouse model of epileptic seizures [25].

The anti-HIV drug efavirenz (EFV) is a first-generation non-nucleoside reverse transcriptase inhibitor [26] and is currently the best-studied small molecule activator of CYP46A1 [27]. EFV enhances CYP46A1 activity by binding to the enzyme allosteric site, which is spatially distinct from the enzyme active site [28]. Only a small dose of EFV (0.1 mg/kg of body weight per day in mice and 50–200 mg/daily in humans) is required to activate CYP46A1 in the brain [11,16,27,29]. In addition, EFV-treated 5XFAD mice, an AD model, had enhanced brain cholesterol turnover, increased sterol flux through the plasma membranes, and altered physico-chemical properties of plasma membranes. Also, there were increased levels of brain acetyl-CoA and acetylcholine (Ach), altered gene expression, synaptic L-Glu release, abundance of synaptic proteins, and protein phosphorylation as well as decreased amyloid β (Aβ) load, either in the whole brain or specific brain regions. Accordingly, either individually or collectively, these treatment outcomes likely contribute, at least in part, to behavioral improvements in EFV-treated animals [11,16,30,31]. However, at higher doses (>0.22 mg/kg of body weight per day in mice) EFV inhibits CYP46A1 by binding to the enzyme active site, i.e., prevents cholesterol from entering the site of catalysis [27].

Only the *(S)*-isomer of EFV inhibits the reverse transcriptase enzyme of HIV [32], whereas both *(S)*- and *(R*)-EFV activate purified CYP46A1 in vitro [33]. The naturally occurring metabolites of *(S)*-EFV (mono (7- and 8-) and di (7,8- and 8,14-) hydroxylated) are also not pharmacologically active toward HIV [34], whereas their *(S)* isoforms or racemic (*rac*) mixtures activate purified CYP46A1 in vitro [33,35]. The maximal in vitro CYP46A1 activation with EFV hydroxymetabolites is at least 1.4-fold higher than that with *(S)*- and *(R*)-EFV and, at high metabolite concentrations, there is no CYP46A1 inhibition like what occurs with *(S)*- or *(R*)-EFV [33,35]. We established that CYP46A1 has two allosteric sites, one for activation by EFV and the other for activation by various neurotransmitters (L-Glu, Ach, and other) [33,35]. We called these two sites EFV- and L-Glu-binding, respectively, and showed that when activators bind to each site simultaneously, their effect on CYP46A1 activity is synergistic [33,35]. We established that EFV monohydroxy metabolites can bind to both CYP46A1 allosteric sites, whereas 7,8-dihydroxy EFV [7,8diOH EFV, either *(S)* or (*rac*)] only interacts with EFV-binding sites, and 8,14-dihydroxy EFV [8,14diOH EFV, either *(S)* or (*rac*)] binds only to the L-Glu-binding site [31]. 

The in vitro activation and binding data raised a question regarding whether EFV hydroxymetabolites can activate CYP46A1 in vivo and lead to brain effects that are similar or even better than those of *(S)*-EFV. We already evaluated (*rac*)-8,14diOH EFV (called 8,14diOH EFV hereafter) on 5XFAD mice and obtained results suggesting that 8,14diOH EFV and *(S)*-EFV should be given to 5XFAD mice simultaneously to test whether such a co-administration will enhance the therapeutic effects of *(S)*-EFV [31]. Herein, (*rac*)-7,8diOH EFV (called 7,8diOH EFV hereafter) was investigated to compare its brain effects with those of 8,14diOH EFV. We established that at a dose (0.1 mg/kg of body weight per day), delivery route (in drinking water), and treatment duration (6 months, from 3 to 9 months of age) which were similar to treatments with 8,14diOH EFV and *(S)*-EFV [16,31], 7,8diOH EFV also activated CYP46A1 in the brain of 5XFAD mice. However, the extent of the enzyme activation and other therapeutically relevant effects were not as pronounced as with *(S)*-EFV and 8,14diOH EFV. These findings suggest that, of the two EFV dihydroxy metabolites evaluated so far, it is 8,14diOH EFV rather than 7,8diOH EFV that should be tested for in vivo co-administration with *(S)*-EFV in the future.

## 2. Results

### 2.1. Behavioral Tests

**Figure 1:**
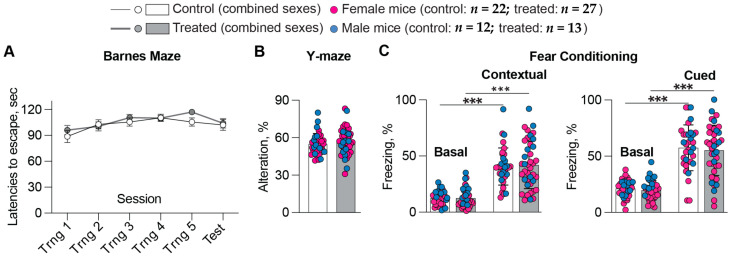
7,8-DihydroxyEFV effect on performance of 5XFAD mice in (**A**), Barnes maze; (**B**), Y-maze; and (**C**), fear conditioning. Data represent the mean ± SEM of the individual measurements (22 control female mice, 12 control male mice, 27 treated female mice, and 13 treated male mice). Two-way repeated measures ANOVA with Bonferroni correction was used to determine if there were sex-based differences within each group. If no sex-based differences were found, then data for female and male mice within each group were combined, and a two-tailed unpaired Student’s *t*-test was used to assess statistical significance. ***, *p* ≤ 0.001. Trng, training.

Cognitive improvements are a goal of any AD therapy. Therefore, before euthanasia, 5XFAD mice were subjected to memory tasks, which were the same as in 8,14diOH EFV treatment: the Barnes maze test, which evaluates the long-term spatial memory; the Y-maze test, which reflects the short-term spatial memory; and fear conditioning tests, contextual and cued, which pertain to emotional memory and associative learning, respectively [31]. No difference in behavioral performance (latency to escape platform, Figure 1A, and total number of errors before entering the escape hole, data not shown) was observed in 7,8diOH EFV-treated vs. control 5XFAD mice of both sexes in the Barnes maze. Similarly, the test results were not improved in Y-maze (Figure 1B). Finally, there were no changes between the treated and control groups in the contextual and cued fear conditioning, even though both groups were able to learn during training, as indicated by a lower percent of basal freezing (Figure 1C). Thus, treatment with 7,8diOH EFV did not seem to improve all memory types, which are impaired in 5XFAD mice [36,37].

### 2.2. Brain Aβ Content

**Figure 2:**
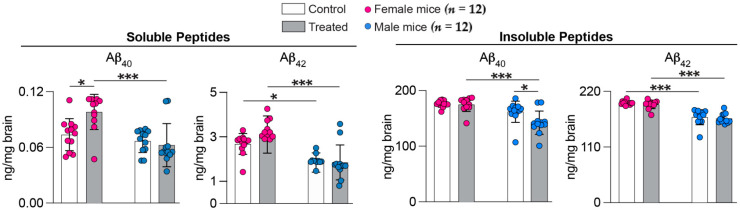
7,8-DihydroxyEFV effect on the Aβ content in the brain of 5XFAD mice. Data represent the mean ± SD of the individual measurements (12 female and 12 male animals per group). *, *p* ≤ 0.05; ***, *p* ≤ 0.001 as assessed by two-way ANOVA with Tukey’s multiple comparison test.

Lowering the brain load of Aβ plaques, composed of insoluble Aβ peptides, is a desirable therapeutic outcome in addition to cognitive improvements. The overall brain Aβ load was assessed by the measurements of Aβ_40_ and Aβ_42_ peptides in brain homogenates (Figure 2). The content of soluble Aβ_40_ peptides was increased in treated vs. control female mice (133%, 0.098 vs. 0.073 ng/mg brain) and was the same in both groups of male mice (0.063 vs. 0.066 ng/mg brain). The content of insoluble Aβ_40_ peptides was the same in treated vs. control female mice (175 vs. 176 ng/mg brain) and was decreased in treated vs. control male mice (88%, 142 vs. 162 ng/mg brain). In both sexes, the treatment did not alter the levels of the Aβ_42_ species: the soluble Aβ_42_ levels were 3.09 vs. 2.67 ng/mg brain in the treated vs. control female mice and 1.85 vs. 1.82 ng/ng brain in treated vs. control male mice. The insoluble Aβ_42_ levels were 194 vs. 197 ng/mg brain in treated vs. control female mice and 166 vs. 168 ng/ng brain in treated vs. control male mice. Thus, only the Aβ_40_ peptides were affected by 7,8diOH EFV treatment, and their changes were sex-specific, benefiting only male 5XFAD mice.

### 2.3. Brain Sterol Profiles and CYP46A1 Expression

**Figure 3:**
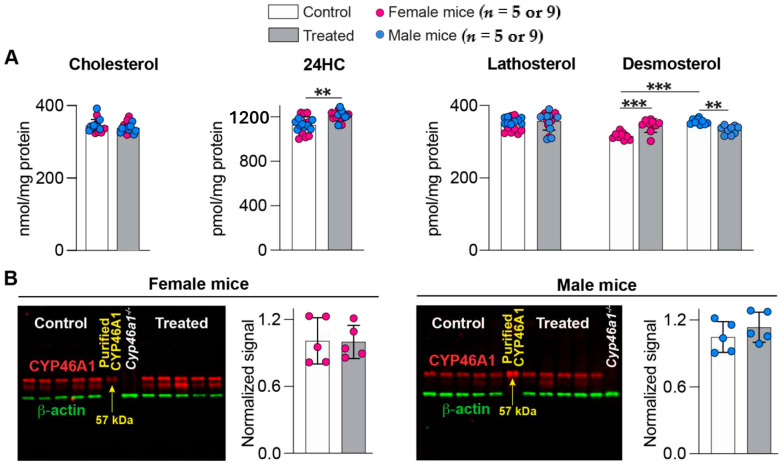
7,8-DihydroxyEFV effect on sterol content and CYP46A1 expression in the brain of 5XFAD mice. (**A**) Sterol quantifications. Data represent the mean ± SD of the individual measurements (9 female and 9 male 5XFAD mice per group). Two-way repeated measures ANOVA with Bonferroni correction was used to determine if there were sex-based differences within each group. If no sex-based differences were found, then data for female and male mice within each group were combined, and a two-tailed unpaired Student’s *t*-test was used to assess statistical significance. Otherwise, data for female and male mice were presented separately. **, *p* ≤ 0.01; ***, *p* ≤ 0.001. (**B**) Representative Western blots (left panels) evaluating CYP46A1 expression in brain homogenates. Each lane, except that with purified recombinant CYP46A1 (used as a positive control), is a sample from an individual animal (five female and five male mice per group); the brain homogenate from a *Cyp46a1^-/-^* mouse was used as a negative control. All Western blots were repeated at least three times. Protein expression quantification (right panels). Within a group, the protein expression in each sample was first normalized to the β-actin expression, and the mean value was calculated. This mean value was then normalized to the mean value of the protein expression in control 5XFAD mice (taken as one), and the data were presented as the mean ± SD. No statistically significant difference was found between control and treated groups of both sexes as assessed using a two-tailed, unpaired Student’s test.

CYP46A1 activation in the brain was assessed by the measurements of different sterols and CYP46A1 expression. Cholesterol is CYP46A1 substrate and 24HC is the CYP46A1 product, whereas lathosterol and desmoterol are cholesterol precursors, which reflect cholesterol biosynthesis in neurons and astrocytes, respectively [38]. In both sexes of 7,8diOH EFV-treated vs. control 5XFAD mice, the cholesterol levels remained the same (339 vs. 346 nmol/mg protein), and the 24HC levels were modestly elevated (108%, 1211 vs. 1127 pmol/mg protein) (Figure 3A). Both sexes had unaltered lathosterol levels that only showed a trend at *p* = 0.07 to a 103% increase. The desmosterol levels were changed: modestly increased in female mice (109%, 344 vs. 317 pmol/mg protein) and decreased (94%, 333 vs. 355 pmol/mg protein) in male mice. In both sexes, the CYP46A1 expression was not affected by 7,8diOH EFV treatment (Figure 3B); therefore, an increase in the 24HC levels in 7,8diOH EFV-treated vs. control 5XFAD mice was likely due to CYP46A1 activation by 7,8diOH EFV. Thus, like (S)-EFV and 8,14diOH EFV [16,31], 7,8diOH EFV seemed to reach CYP46A1 in the brain and enhance the enzyme activity. However, this CYP46A1 activation was only modest. 

### 2.4. Brain Acetyl-CoA Levels

**Figure 4:**
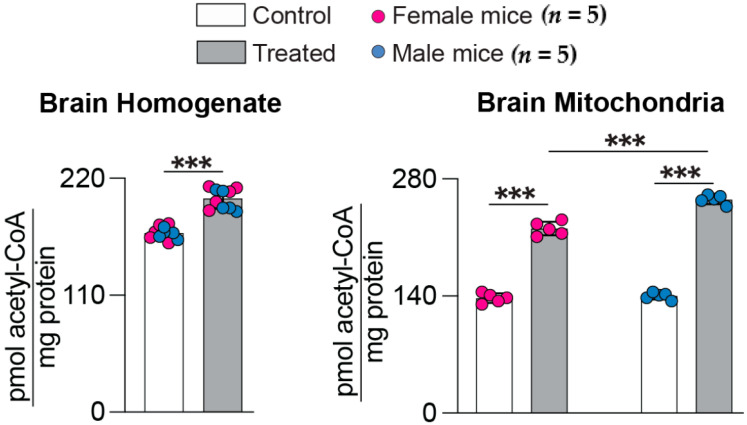
7,8-DihydroxyEFV effect on the acetyl-CoA levels in the brain of 5XFAD mice. Data represent the mean ± SD of the individual measurements (five female and five male mice per group). Two-way repeated measures ANOVA with Bonferroni correction was used to determine if there were sex-based differences within each group. If no sex-based differences were found, then data for female and male mice within each group were combined, and a two-tailed unpaired Student’s *t*-test was used to assess statistical significance. Otherwise, data for female and male mice were presented separately. ***, *p* ≤ 0.001.

Acetyl-CoA is a cofactor, required for many cellular processes, including energy production and the synthesis of Ach from choline [39]. The brain levels of acetyl-CoA were increased in 5XFAD mice after treatments with *(S)*-EFV and 8,14diOH EFV [30,31]. Therefore, we investigated whether this is the case with 7,8diOH EFV by measuring total (in brain homogenates) and mitochondrial acetyl-CoA pools. Both pools were increased in 7,8diOH EFV-treated vs. control 5XFAD mice (Figure 4). In brain homogenates, there was a 120% increase (201 vs. 168 pmol/mg protein in treated vs. control mice of both sexes). In the mitochondrial fraction, the increases were higher: 160%, 220 vs. 137 pmol/mg protein in treated vs. control female mice, and 182%, 255 vs. 140 pmol/mg protein in treated vs. control male mice. Thus, *(S)*-EFV and both its dihydroxymetabolites increased the acetyl-CoA levels in the brains of 5XFAD mice.

### 2.5. Brain Ach Levels

**Figure 5:**
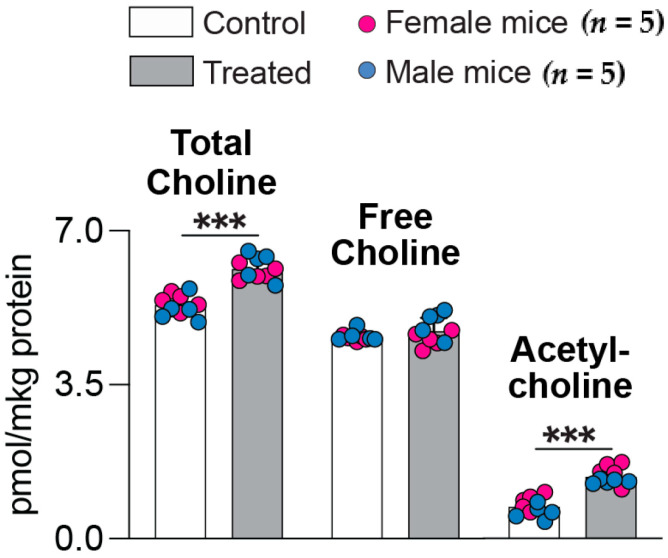
7,8-DihydroxyEFV effect on the Ach levels in the brain of 5XFAD mice. Data represent the mean ± SD of the individual measurements (five female and five male mice per group). ***, *p* ≤ 0.001 as assessed by two-way ANOVA with Tukey’s multiple comparison test.

Ach is a neurotransmitter produced by cholinergic neurons, which selectively degenerate in AD, thus leading to a decrease in the Ach levels [40]. Similar to acetyl-CoA, the Ach levels were increased in the brain of 5XFAD mice after treatments with *(S)*-EFV and 8,14diOH EFV [31]. Therefore, this neurotransmitter was quantified in the present work as a difference between the content of total (free choline plus Ach after the hydrolysis of the acetyl group) and free choline (Figure 5). In both sexes of 7,8diOH EFV-treated vs. control mice, the levels of total choline were increased (115%, 6.1 vs. 5.3 pmol/μg protein), whereas the levels of free choline remained unchanged (4.7 vs. 4.6 pmol/μg protein). Accordingly, the Ach levels were increased (200%, 1.4 vs. 0.7 pmol/μg protein) after the treatment. Thus, not only the acetyl-CoA levels but also the Ach levels were increased after treatments with *(S)*-EFV and both its dihydroxymetabolites.

### 2.6. In Vitro Incubations of CYP46A1 with Two Activators

**Figure 6:**
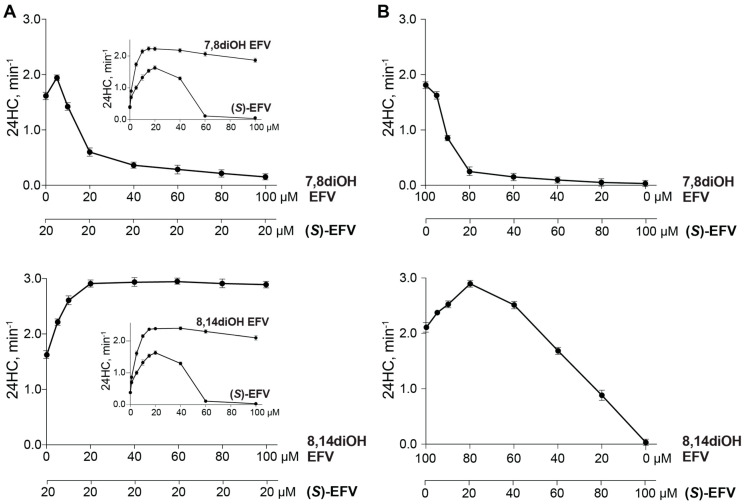
CYP46A1 activation in the in vitro co-incubations with *(S)*-EFV and a dihydroxy EFV metabolite. (**A**) *(S)*-EFV was used at a fixed (20 μM) concentration, and the concentration of a dihydroxyEFV metabolite varied from 0 to 100 μM. (**B**) Both *(S)*-EFV and a dihydroxy EFV metabolite were used at varied concentrations, increasing from 0 to 100 μM for *(S)*-EFV and decreasing from 100 to 0 μM for a dihydroxy EFV metabolite. CYP46A1 activity represents nanomoles of 24-hydoxycholesterol (24HC) per nanomole of CYP46A1 per min. The results are the mean ± SD of the three independent experiments.

Purified recombinant CYP46A1 and the in vitro reconstituted system were used to gain insight into the absolute amounts as well as the ratios between *(S)*-EFV and a dihydroxyEFV metabolite if they are co-administered in vivo. In the first set of experiments, *(S)*-EFV was used at a fixed concentration (20 μM) that elicts a maximal enzyme activation in vitro [35] and varying concentrations of a dihydroxyEFV metabolite, either 7,8diOH EFF or 8,14diOH EFV (Figure 6A). Under these co-incubation conditions, there was CYP46A1 inhibition at high 7,8diOH EFV concentrations, and no inhibition at high 8,14diOH EFV concentrations. Notably, the maximal CYP46A1 activation in the co-incubations of 8,14diOH EFV and *(S)*-EFV) was higher (3.0 min^−1^) than that in the incubations with individual activators (up to 2.4 min^−1^, Figure 6A inset). In the second set of experiments, the concentrations of *(S)*-EFV and a dihydroxy EFV metabolite varied, so that, in each co-incubation, the total compound concentration did not exceed 100 μM. Under these co-incubation conditions, increasing concentrations of *(S)*-EFV inhibited CYP46A1 at all concentrations of 7,8diOH EFV, whereas in the case of 8,14diOH EFV, there were activatior concentrations (60–100 μM) that still activated CYP46A1 (Figure 6B). Thus, collectively, the two co-incubation conditions suggested that, for in vivo co-administrations, 8,14diOH EFV should be used and the *(S)*-EFV dose should remain the same as in the individual treatment, while the 8,14diOH EFV dose can vary from that of *(S)*-EFV to a 4-fold higher, i.e., represent from 1:1 to 1:4 (*w*/*w*) *(S)*-EFV to metabolite ratio.

## 3. Discussion

Herein, we continued our in vivo evaluations of EFV dihydroxy metabolites and focused on the brain effects of 7,8diOH EFV. We used the same mouse model (5XFAD mice) and treatment paradigm as in the investigations of *(S)*-EFV and 8,14diOH EFV and selected the endpoints that could be of therapeutic relevance [11,16,30,31]. In addition, we conducted in vitro studies of CYP46A1 activation by 7,8diOH or 8,14diOH EFV in co-incubations with *(S)*-EFV. Thus, we can now compare the effects of the two EFV dihydroxy metabolites both between each other and with those of *(S)*-EFV, and use the knowledge obtained for developing treatments that could lead to a higher CYP46A1 activation in vivo and, perhaps, even better therapeutic outcomes.

We established that 7,8diOH EFV elicited a lower CYP46A1 activation in 5XFAD mice than 8,14diOH EFV and *(S)*-EFV (108% vs. 119%, Table 1) as indicated by brain levels of the CYP46A1 product 24HC (Figure 3A) and unchanged CYP46A1 expression in the brain (Figure 3B). This was unexpected because 7,8diOH EFV binds to the same allosteric site on the enzyme surface as *(S)*-EFV [33] and has chemical properties similar to those of 8,14diOH EFV. The total polar surface area of 7,8diOH EFV is 78.8 Å^2^ and the predicted log p value is 4.0 with both properties suggesting good CNS availability [41]. Also, while no data are available for *(S)*-7,8diOH EFV, *(S)*-EFV and its hydroxymetabolites [*(S)*-7OH, *(S)*-8OH, and *(S)*-8,14diOH)] are known to be highly protein-bound in the plasma (>99%). Nevertheless, these compounds are detected in the cerebrospinal fluid [34,42,43,44,45], an indication of presence in the brain. Accordingly, it is likely not a delivery of 7,8diOH EFV to the brain, as the metabolite dose was the same as that of 8,14diOH EFV, but its availability to CYP46A1 in brain neurons that underlie a lower enzyme activation by this EFV metabolite.

Apparently, a modest CYP46A1 activation by 7,8diOH EFV was not sufficient to increase cholesterol turnover in brain neurons as both sexes had only a trend to a minor increase in lathosterol levels (Table 1). However, in brain astrocytes, cholesterol turnover may have been increased in female mice but not male mice, as desmosterol levels were modestly increased and decreased, respectively (Table 1). Accordingly, sex-specific changes were observed in the desmosterol levels in 7,8diOH EFV-, 8,14diOH EFV-, and *(S)*-EFV-treated 5XFAD mice, yet in the latter two, desmosterol levels were never decreased and either did not change or were increased (Table 1). 

Similar to *(S)*-EFV and 8,14diOH EFV, CYP46A1 activation by 7,8diOH EFV was sufficient to increase the acetyl-CoA and Ach levels, although increases in the former of both total and mitochondrial pools were lower than those with *(S)*-EFV and 8,14diOH EFV (Table 1). Thus, of the three compounds tested so far, *(S)*-EFV elicited the highest increases in the acetyl-CoA levels in 5XFAD mice, and animals of both sexes had improved performance in the Morris Water maze test (Table 1). In 8,14diOH-EFV treatment, increases in the acetyl-CoA pools in male mice were comparable with those in *(S)*-EFV-treated mice and were higher than those in 8,14diOH-EFV-treated female mice; it is therefore probably that only the treated male mice had improved performance in the Barnes maze test (Table 1). Finally, 7,8diOH EFV treatment led to the lowest increases in the total and mitochondrial acetyl-CoA levels, and neither animal sex showed improvements in the Barnes maze test (Table 1). Hence, it is feasible that increases in the acetyl-CoA pools could correlate with improved performance in the tests for the long-term spatial memory. Obviously, more studies are required to confirm the suggested acetyl-CoA-behavioral performance correlation.

The present work, along with the previous 8,14diOH-EFV treatment, pointed to a possible dihydroxy EFV-specific effect on insoluble Aβ load in brain homogenates. In 8,14diOH EFV-treated mice, a decrease was in the levels of insoluble Aβ_40_ peptides in both sexes. In 7,8diOH EFV-treated mice, a decrease was in the levels of insoluble Aβ_42_ peptides and at least in one sex (male mice) (Table 1). In *(S)*-EFV-treated mice of both sexes, there was a decrease in the levels of insoluble Aβ peptides (Aβ_40_ and Aβ_42_) in brain homogenates as well, but only when the treatment started at 1 month of age, before the appearance of Aβ plaque [11]. However, when the treatment started at 3 months of age, as with 7,8diOH EFV and 8,14diOH EFV, *(S)*-EFV did not decrease the brain Aβ load globally (Table 1) but rather locally in the mouse cortex and hippocampus [16]. Thus, when the treatment starts at 3 months of age, i.e., when Aβ plaques are already present in the 5XFAD brain, the dihydroxy EFV metabolites have a stronger Aβ lowering effect than *(S)*-EFV. This is an important finding that needs to be further investigated and kept in mind while developing an optimal EFV treatment strategy. 

No information is currently available about brain concentrations of *(S)*-EFV and its hydroxymetabolites after *(S)*-EFV dosing, and there did not seem to be any in vivo treatments with EFV dihydroxymetabolites prior to the present work and our previous study [31]. However, in the case of *(S)*-EFV administration, it is known that *(S)*-EFV could accumulate intracellularly (e.g., in peripheral blood mononuclear cells) and in the brain [46,47,48], and that there could be brain metabolism of *(S)*-EFV and its hydroxymetabolites [42,43,49]. For example, in the cerebrospinal fluid, *(S)*-7OH EFV was found to be mainly glucuronidated, and *(S)*-8OH EFV was either glucuronidated or sulfated [42,43]. Of pertinence is an in vitro study in which brain microsomes from different species (mouse, macaque, and human) were incubated with (*rac*)-EFV [49], i.e., a mixture of *(S)*- and (*R*)-EFV, the latter being metabolized to 8OH EFV by CYP2B6 in vitro at a rate 10 times lower than that of *(S)*-EFV [50]. Nevertheless, under the experimental conditions used, brain microsomes from all three species produced 8OH EFV and its glucuronide as well as 8,14diOH EFV glucuronide, whereas 7OH EFV and 8,14diOH EFV were not detected [49]. Additional studies in cell culture demonstrated that the primary astrocytes, striatal neurons, or cortical neurons did not exhibit any metabolic activity toward (*rac*)-EFV, and only the microglial cells converted (*rac*)-EFV to 8OH EFV [49]. 

In addition to local metabolism, the brain concentration of *(S)*-EFV and its hydroxymetabolites could depend on the amounts delivered by the systemic circulation. For example, in human subjects treated with the 600 mg/day *(S)*-EFV dose, the total plasma concentrations (protein-free plus protein-bound forms) were found to be 2170–3350 ng/mL for *(S)*-EFV, 315–2160 ng/mm for 8OH EFV, 8.8–225 ng/mL for 7OH EFV, and 10.2 ng/mL for 8,14OH EFV, but no information is available about plasma concentrations of 7,8diOH EFV [34,42,44,45,51,52]. Thus, *(S)*-EFV treatment could lead to very different 7,8diOH EFV and 8,14diOH EFV concentration in the brain and, therefore, availability to CYP46A1 in brain neurons. Accordingly, it is reasonable to test in the future a simultaneous administration of *(S)*-EFV and its dihydroxy metabolite, which will likely lead to higher metabolite amounts delivered to the brain than upon dosing with only *(S)*-EFV. 

The current work and our previous study [31] collectively suggest that 8,14diOH EFV rather than 7,8diOH EFV should be used for a co-administration, even though both activated CYP46A1 in the brain of 5XFAD mice at the same 0.1 mg/kg body weight dose as *(S)*-EFV, and both elicited therapeutically relevant changes in the brain levels of Aβ, acetyl-CoA, and Ach (Table 1). However, quantitatively, the effects are better and more sex-independent in the case of 8,14diOH EFV than 7,8diOH EFV, perhaps because they bind to different allosteric sites. Of consideration is that, both in vitro and in vivo, *(S)*-EFV has a rather narrow range of concentrations/doses that activate CYP46A1 [27], even though the mouse dose (0.1 mg/kg body weight) that was used in our treatments was ~86 times lower than that (600 mg/day) given to HIV-positive individuals. This is in contrast to 7,8diOH EFV and 8,14diOH EFV, which do not inhibit CYP46A1, at least in vitro [31,33]. The results of in vitro testing of different co-incubation conditions of CYP46A1, 8,14diOH EFV, and *(S)*-EFV are also encouraging as they showed that, under certain activator concentrations, there is an even higher CYP46A1 activation than that which occurs with the individual activators (Figure 6). Accordingly, we suggest future research into simultaneous administration of *(S)*-EFV and 8,14diOH EFV to 5XFAD mice, each at a 0.1 mg/dose, and evaluate whether this treatment will lead to the synergistic activator effects, i.e., will enhance CYP46A1 activation, behavioral improvements and positive changes in the Aβ, acetyl-CoA, and Ach levels.

In summary, we treated 5XFAD mice for 6 months with 7,8diOH EFV at the 0.1 mg/kg body weight dose and evaluated animal behavioral performance in different tasks along with measurements of the brain Aβ load, sterol profiles, CYP46A1 expression, acetyl-CoA, and Ach levels. The results were compared to those obtained previously in treatments with 8,14diOH EFV and *(S)*-EFV. This comparison suggested that 7,8diOH EFV is not as effective in CYP46A1 activation in vivo as *(S)*-EFV or its 8,14-dihydroxy metabolite, and that a co-administration of *(S)*-EFV and 8,14diOH EFV are warranted to ascertain whether there could be a higher CYP46A1 activation in vivo and/or stronger clinically relevant activation effects than after *(S)*-EFV dosing.

## 4. Materials and Methods

### 4.1. Animals and Treatment

5XFAD mice were hemizygous for the mutant human amyloid precursor protein 695 (K670N, M671L, I716V, V717I) and mutant human presenilin 1 (M146L and L286V) [36]. These mice were generated by crossing hemizygous 5XFAD male mice on the B6SJL background (the Jackson Laboratory, stock No: 34840, Bar Harbor, ME, USA) and wild type B6SJL females (the Jackson Laboratory, stock No: 100012). The latter were bred out of the *Pde6b^rd1^* mutation, which leads to early onset severe retinal degeneration and, hence, blindness [53]. Only the F1 generation was used. Animal treatment with *(rac)*-7,8-dihydroxyEFV (Toronto Research Chemicals, #D452800, Toronto, ON, Canada) was the same as with *(S)*-EFV [16] and *(rac)*-8,14-dihydroxyEFV [31]. Briefly, *(rac)*-7,8-dihydroxyEFV was administered at a 0.1 mg/kg body weight/day dose in drinking water supplemented with 0.0004% Tween 80, and aqueous 0.0004% Tween 80 was given to control animals. The treatment duration was 6 months, namely from 3 to 9 months of age. Mice were maintained in a temperature and humidity-controlled environment with 12 h light/12 h dark cycle in cages with water and food ad libitum. Mice drank ~5.6 mL of water per day, the same amount as in treatments with *(S)*-EFV and 8,14diOH EFV. All animal experiments (the protocol # 2014-0154) were approved by the Case Western Reserve University’s Institutional Animal Care and Use Committee and conformed to recommendations by the American Veterinary Association Panel on Euthanasia.

### 4.2. Behavioral Tests

The tests and their order were the same as in treatment with *(rac)*-8,14-dihydroxyEFV [31]: days 1–3 and 5—the Barnes maze test; day 8—the Y-maze test; and days 9–10—the fear conditioning memory tests. On day 1, mice were acclimated to the Barnes maze, and on days 2 and 3, animals had training sessions 1–3 and 4–5, respectively. During training, mice used visual cues on the floor and wall of the maze to locate the escape hole and leave a brightly lit arena. On day 5, mice were given a maximum of 120 sec to locate the escape hole, and latency to discover the hole and time spent in the same circular quadrant as the escape hole were recorded for analysis [31]. In the Y-maze, which had arms of equal length, mice were allowed 8 min of exploration, followed by recording of the number of entries, order of arm entries, and spontaneous alterations (entry into three arms non-consecutively), and the calculation of the percentage of entries that were spontaneous alterations [31]. The ANY-maze software (Stoelting Co., Wood Dale, IL, USA) was used to analyze the Barnes maze and Y-maze tests. In a chamber for the contextual and cued fear memory tests, mice were presented with one 30 s audible tone that co-terminated with a mild foot shock for the tone-shock pairing, and then were returned to their home cage. In 24 h, mice were placed in the same unaltered chamber for 5 min of observation (contextual memory), and 5 h later were placed in the chamber with altered tactile, visual, and olfactory cues (cued memory). In this altered chamber, mice were exposed to two audible tones identical to those presented during the initial tone-shock pairing, and the freezing behavior was recorded, i.e., the cessation of all movement except for breathing. Freezing was used to index fear memory defined as a percent of freezing time during the experiment [31]. The VideoFreeze software (version 3.0.0, Med Associates, Inc., Fairfax, VT, USA) was used to analyze the contextual and cued memory tests.

### 4.3. Brain Processing 

Mice were euthanized in the morning after overnight fasting. Subsequent brain processing was as described [11,31], and namely included brain isolation, rinsing in cold phosphate buffer saline, blotting, removal of the brain stem and olfactory bulb, and dissection along the midline to obtain two hemispheres. The left hemisphere was used for the preparation of 10% (*w*/*v*) brain homogenate in 50 mM potassium phosphate buffer, pH 7.2, containing 300 mM sucrose, 0.5 mM dithiothreitol, 1 mM EDTA, and a cocktail of protease inhibitors (cOmplete, Sigma-Aldrich, #11697498001, St. Louis, MO, USA). This homogenate was used for the quantification of sterols, acetyl-CoA, and Ach as well as Western blots. The cerebellum was removed from the right hemisphere, which was then used for the preparation of 10% (*w*/*v*) brain homogenate in 20 mM Tris-HCl buffer, pH 7.4, containing 250 mM sucrose, 0.5 mM EDTA, 0.5 mM EGTA, and a cocktail of protease inhibitors. This homogenate was used for soluble and insoluble Aβ peptide quantification.

### 4.4. Brain Aβ Content

Diethylamine (0.2%) and formic acid (70%) were used for extraction of soluble and insoluble Aβ peptides, respectively, from brain homogenates as described [11], which were then quantified by ELISA kits for the Aβ_40_ and Aβ_42_ species (ThermoFisher Scientific, #KHB3482 and #KHB3441, respectively, Waltham, MA, USA) according to the manufacturer’s protocol.

### 4.5. Brain Sterol Profiles 

Cholesterol, lathosterol, and desmosterol were quantified as total sterols (free plus esterified sterol), and 24HC was quantified as free sterol as described [16] by isotope dilution gas chromatography-mass spectrometry using deuterated sterol analogs as internal standards. 

### 4.6. CYP46A1 Western Blots

Western blots were carried out as described [16,31] using in-house-generated mouse monoclonal IgG (clone 9C5-B2-A1, 1:500 dilution) against CYP46A1 and the secondary goat anti-mouse IgG (Li-Cor, 680RD, Lincoln, NE, USA, 1:10,000 dilution). The primary antibody for β-actin was polyclonal rabbit IgG (Abcam, ab8227, Cambridge, MA, USA, 1:1000 dilution), and the secondary antibody was goat anti-rabbit IgG (Li-Cor, IRDye 800 CW, Lincoln, NE, USA, 1:10,000 dilution).

### 4.7. Brain Acetyl-CoA Levels

The acetyl-CoA content was measured using the acetyl-CoA assay kit (Sigma-Aldrich, St. Louis, MO, USA, #MAK039) in brain homogenates and mitochondria, which were prepared as described [30,31,54]. Briefly, mitochondria were isolated by the Percoll density gradient (23–40%), then treated with 0.02% digitonin, and washed by centrifugation with 10 mM Tris-HCl buffer, pH 7.4, containing 166 mM sucrose, and 1 mM EDTA, to remove digitonin. The mitochondria purity after this procedure was 99%, as indicated by the relative intensity of the Western blot signals for ATP5A and calnexin (markers for mitochondria and endoplasmic reticulum, respectively) [30].

### 4.8. Brain Ach Levels

Free and total choline were quantified by the choline/acetylcholine quantification kit (Sigma-Aldrich, #MAK056) according to the manufacturer’s protocol.

### 4.9. In Vitro CYP46A1 Incubations with Two Activators

Expression of CYP46A1 and cytochrome P450 oxidoreductase in *Escherichia coli,* protein purification, and CYP46A1 activity measurements were as described [35,55]. Briefly, enzyme assays were carried for 30 min at 37 °C in 1 mL of 50 mM potassium phosphate buffer, pH 7.2, containing 100 mM NaCl, 40 µM cholesterol, and 40 μg/mL L-α-1,2-dilauroyl-*sn*-glycero-3-phosphocholine. The activator concentrations varied and are described in the Figure 6 legend. The protein concentrations were 0.5 µM CYP46A1, 1.0 µM cytochrome P450 oxidoreductase, 2 units of catalase, and an NADPH-regenerating system (1 mM NADPH, 10 mM glucose-6-phosphate, and 2 units of glucose-6-phosphate dehydrogenase). Sterols were extracted with 5 mL of dichloromethane containing 3 nmol of 24-hydroxy-[25,26,26,26,27,27,27-^2^H_7_]-cholesterol as an internal standard, then processed and analyzed by gas chromatography-mass spectrometry, as described [16]. 

### 4.10. Statistics

Data from all available animals were used; the sample size (n) is indicated in each figure legend. All data represent the mean ± SD of the individual measurements; only in behavioral tests, the mean ± SEM was used. Data were analyzed by unpaired Student’s t test assuming a 2-tailed distribution, two-way repeated measures ANOVA with Bonferroni correction, or two-way repeated measures ANOVA with Tukey’s multiple comparison test. Statistical significance was defined as *, *p* ≤ 0.05; **, *p* ≤ 0.01; ***, *p* ≤ 0.001.

## Figures and Tables

**Table 1:** A comparison of relative (%) changes in treated vs. control 5XFAD mice when either different dihydroxymetabolites of EFV or *(S)*-EFV were used.

Molecule or Test		7,8diOH EFV ^1^	8,14diOH EFV ^1^	*(S)*-EFV ^1^
Cholesterol	F ^2^	NC ^3^	106	NC
	M ^2^			
24HC	F	108	118	118
	M			
Lathosterol	F	(103%, *p* = 0.07)	120	109
	M			
Desmosterol	F	109	NC	108
	M	94	107	NC
Total Acetyl-CoA	F	120	177	nm
	M		231	259
Mitochondrial Acetyl-CoA	F	160	305	nm
	M	182	357	402
Ach	F	200	233	140
	M		300	240
Barnes (dihydroxy EFV) or Morris Water (*(S)*-EFV) Maze	F	NC	NC	Improved
	M		Improved	
Y-maze	F	NC	NC	NC
	M	NC		
Fear Conditioning Contextual	F	NC	NC	Improved
	M	NC		
Fear Conditioning Cued	F	NC	NC	NC
	M	NC		
Insoluble Aβ_40_ (brain homogenate)	F	NC	NC	NC
	M	88%		
Insoluble Aβ_42_ (brain homogenate)	F	NC	85	NC
	M	NC		

^1^ Changes for 7,8diOH EFV are from the present work, and those for *(S)*-EFV and 8,14diOH EFV were taken from [16,30,31], except the brain sterol data for *(S)*-EFV-treated 5XFAD female mice, which were not included in [16] because they were measured after the manuscript publication. ^2^ F, female mice, M, male mice. When no sex-based differences were found, data for female and male mice were combined; otherwise, they were presented separately. ^3^ NC, no change.

## Data Availability

The data presented in this study are available in the article.
